# Targeted Ubiquitination and Degradation of G-Protein-Coupled Receptor Kinase 5 by the DDB1-CUL4 Ubiquitin Ligase Complex

**DOI:** 10.1371/journal.pone.0043997

**Published:** 2012-08-27

**Authors:** Ziyan Wu, Yuejun Chen, Tong Yang, Qinqin Gao, Man Yuan, Lan Ma

**Affiliations:** The State Key Laboratory of Medical Neurobiology and Pharmacology Research Center, Shanghai Medical College and Institutes of Brain Science, Fudan University, Shanghai, China; California Institute of Technology, United States of America

## Abstract

The G protein-coupled receptor kinases (GRKs) phosphorylate agonist occupied G protein-coupled receptors (GPCRs) and desensitize GPCR-mediated signaling. Recent studies indicate they also function non-catalytically via interaction with other proteins. In this study, a proteomic approach was used to screen interacting proteins of GRK5 in MDA-MB-231 cells and HUVEC cells. Mass spectrometry analysis reveals several proteins in the GRK5 immunocomplex including damaged DNA-binding protein 1 (DDB1), an adaptor subunit of the CUL4-ROC1 E3 ubiquitin ligase complex. Co-immunoprecipitation experiments confirmed the association of GRK5 with DDB1-CUL4 complex, and reveal that DDB1 acts as an adapter to link GRK5 to CUL4 to form the complex. Overexpression of DDB1 promoted, whereas knockdown of DDB1 inhibited the ubiquitination of GRK5, and the degradation of GRK5 was reduced in cells deficient of DDB1. Furthermore, the depletion of DDB1 decreased Hsp90 inhibitor-induced GRK5 destabilization and UV irradiation-induced GRK5 degradation. Thus, our study identified potential GRK5 interacting proteins, and reveals the association of GRK5 with DDB1 in cell and the regulation of GRK5 level by DDB1-CUL4 ubiquitin ligase complex–dependent proteolysis pathway.

## Introduction

G protein-coupled receptor (GPCR) kinases (GRKs) are a family of serine/threonine kinases that phosphorylate GPCRs and desensitize GPCR-mediated signaling. GRK-catalyzed receptor phosphorylation leads to the recruitment of beta-arrestins to phosphorylated receptors, induces receptor internalization, and thus down-regulates cellular responses to extracellular signal [1], [2]. Many studies indicate that GRKs are able to phosphorylate a variety of non-GPCR substrates such as synuclein [3], p38 [4], NF-κB1 p105 [5], ezrin [6], arrestin-2 [7], and p53 [8]. It has also been shown that GRKs can regulate signaling pathways via direct interaction with other proteins in a phosphorylation-independent manner. GRK2 is able to interact with Gαq to regulate GPCR signaling [9]. Binding of GRK5 with IκB inhibits NF-κB-mediated transcription [10]. Our earlier research showed that the kinase activity-independent regulation of the cyclin pathway by GRK2 is essential for zebrafish early development [11] and GRK5 acts as a scaffold to promote F-actin bundling and targets bundles to membrane structures to control neuronal morphogenesis [12]. These studies implicate that GRKs, especially GRK5, may exert multiple physiological functions via various mechanisms including those independent of their kinase activities.

Change in GRK protein level has been detected in a variety of human disorders including heart failure, acute myocardial infarction, hypertension, brain ischemia, rheumatoid arthritis, Parkinson’s disease, Alzheimer’s disease and depression [13], suggesting that protein turnover plays a key role in GRK regulation. The regulation of GRK2 turnover has been studied [14], [15], [16], [17], [18]. Mdm2 plays a key role in regulation of GRK2 ubiquitination and degradation [18]. Hsp90 interacts with and stabilizes GRK2 [19]. However, little is known about regulation of other GRK subtypes.

Damaged DNA-binding protein 1 (DDB1) is part of an E3 ligase complex that includes the cullin proteins CUL4A and CUL4B [20]. The DDB1–CUL4 complex is a conserved cullin-RING ubiquitin ligase, that regulates DNA repair [21], [22], [23], [24], replication [25], [26], [27], and transcription [28]. The DDB1–CUL4 complex can also be subverted by pathogenic viruses to benefit viral infection [29]. CUL4 assembles ubiquitin ligase by binding to ROC1, a RING protein, and to DDB1, a triple β propeller adapter protein, which functions as a linker to recruit substrates or substrate receptors to CUL4 E3 ubiquitin ligase [30].

In an effort to identify proteins that interact with GRK5, we used a proteomic approach to isolate GRK5 interacting proteins and identified several proteins in the GRK5 immunocomplex including DDB1. We further demonstrated that DDB1 acts as an adapter to link GRK5 to CUL4-ROC1 E3 ligase complex and regulates GRK5 ubiquitination and degradation.

## Results

### Identification of GRK5-interacting Proteins by Mass Spectrometry

**Figure 1:**
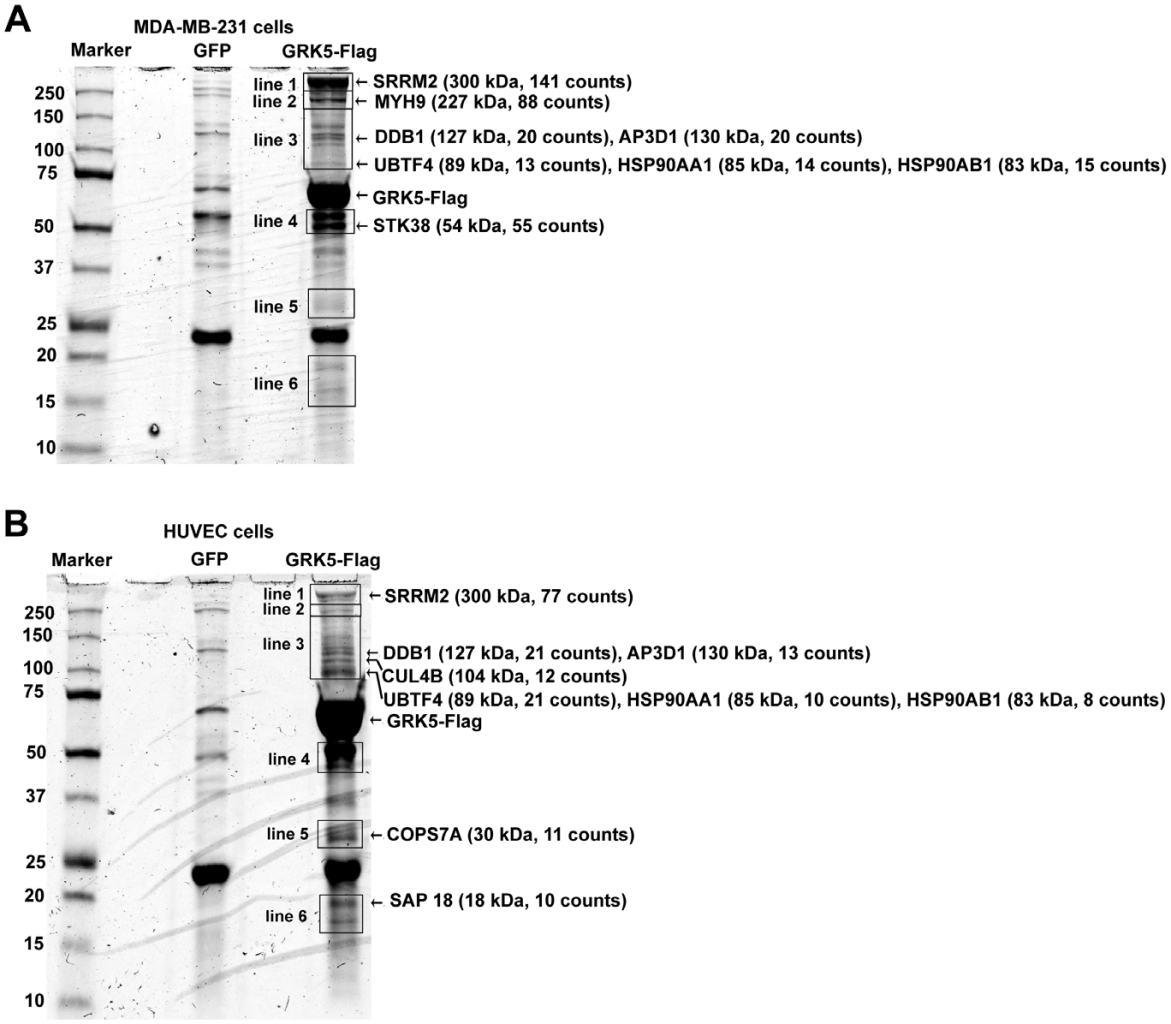
Identification of GRK5 interacting proteins by mass spectrometry. (A) Lysates of MDA-MB-231 cells stably expressing GFP or GRK5-Flag were immunoprecipitated with M2 affinity gel. The complexes were separated on SDS-PAGE and stained with Coomassie Blue. Six pieces of gels were excised and labled as line 1-line 6 as indicated. The corresponding control lane was also excised. These gels were analyzed by mass spectrometry. The major associated proteins identified by mass spectrometry were indicated. (B) Lysates of HUVEC cells transiently expressing GFP or GRK5-Flag were treated as in (A). The gels were analyzed by mass spectrometry. The associated proteins identified by mass spectrometry were indicated.

A proteomic approach was used to identify interacting proteins of GRK5. Flag-tagged GRK5 was affinity purified from MDA-MB-231 cells stably expressing GRK5-Flag. As a control, cells stably expressing GFP were taken through the same procedure. The resulting protein complexes were electrophoresed on a 4–20% gradient polyacrylamide gel and then stained with Coomassie Blue. Protein bands of ∼300 kDa, ∼230 kDa, ∼130 kDa, ∼85 kDa and ∼55 kDa were selectively copurified with Flag-tagged GRK5 but not control beads. The gel bands were excised and analyzed by mass spectrometry as indicated in Figure 1A. The corresponding bands in the control lane were also excised and analyzed by mass spectrometry, and proteins detected in both the control lane and GRK5-immuocomplex lane were excluded from further analysis. This work led to the identification serine/arginine repetitive matrix 2 (SRRM2, 300 kDa), Myosin-9 (MYH9, 227 kDa), AP-3 complex subunit delta 1 (AP3D1, 130 kDa), damage-specific DNA binding protein 1 (DDB1, 127 kDa), heat shock protein Hsp90-alpha (Hsp90AA1, 83 kDa), heat shock protein Hsp90-beta (Hsp90AB1, 85 kDa), upstream binding transcription factor, RNA polymerase 1 (UBTF4, 89 kDa), nucleolin (NCL, 77 kDa) and serine/threonine kinase 38 (STK38, 55 kDa) as the major interacting proteins of GRK5 in MDA-MB-231 cells (Fig. 1A and Table S1). DDB1 functions as a linker to recruit substrates or substrate receptors to CUL4-ROC1 E3 ubiquitin ligase [20]. Interestingly, peptides derived from other components of DDB1-CUL4 complex were also identified in the GRK5 immunoprecipitates by mass spectrometry including CUL4B, which interacts with DDB1 and assembles ubiquitin ligase by binding to ROC1; WD40-repeat protein 22 (WDR22) and glutamate-rich WD40-repeat protein 1 (GRWD1), which contain WD40 repeat domain and constitute the substrate-specific adaptor that recruits substrates to the DDB1-CUL4 complexes through interaction with DDB1; COP9 signalosome complex subunit 7a (COPS7A), component of the COP9 signalosome complex, which cleaves NEDD8 from CUL4 and regulates the CUL4 ubiquitin ligase activity (Table S1 and S3). Similar proteomic approach was carried out in human umbilical vein endothelial Cells (HUVEC) cells transiently transfected with Flag-GRK5. SRRM2, AP3D1, Hsp90, UBTF4 and NCL were also detected in HUVEC cells by mass spectrometry (Fig. 1B, supplemental Table S1, S2 and S4). The DDB1-CLU4 complex proteins including DDB1, CUL4B, WDR22, GRWD1 and COPS7A were all detected in HUVEC cells (Fig. 1B, supplemental Table S1 and S2), and two additional WD40-repeat domain containing proteins were identified in HUVEC cells including WDR12 and WDR82 (Table S2 and S4). However, STK38, which was readily detected in the GRK5 immunocomplex in MDA-MB-231 cells by mass spectrometry, was not detected in the HUVEC cells (Fig. 1A, 1B and Table S1,S2,S3,S4). As we identified several components of DDB1-CUL4 complex in both MDA-MB-231 cells and HUVEC cells by mass spectrometry, we further examined the association of GRK5 with DDB1-CUL4 ubiquitin ligase complex.

### GRK5 Associates with DDB1-CUL4 Ubiquitin Ligase Complex

**Figure 2:**
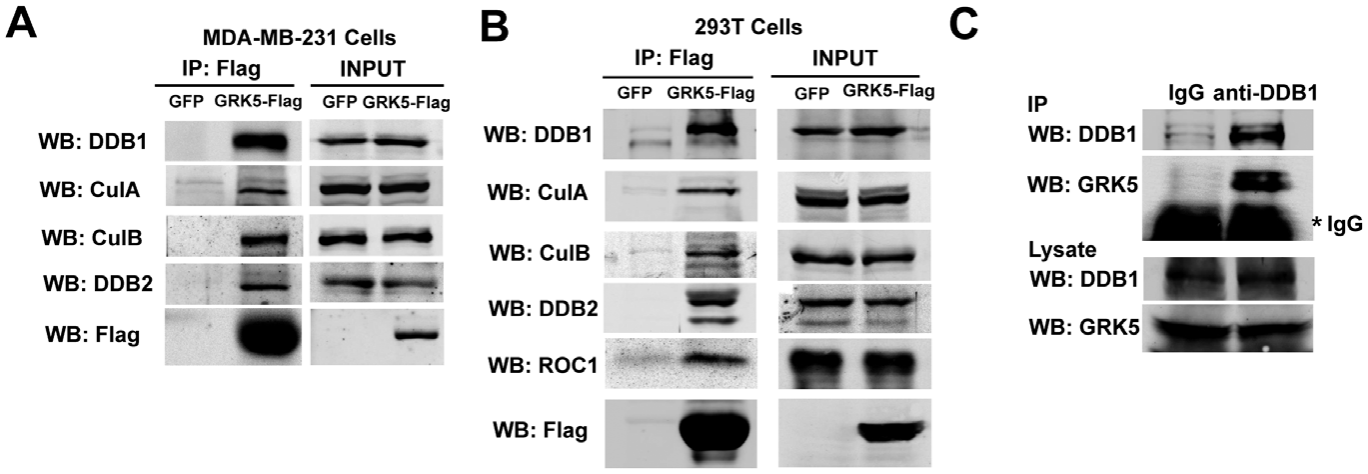
GRK5 associates with DDB1-CUL4 ubiquitin ligase complex. (A,B) MDA-MB-231 cells stably expressing GFP or GRK5-Flag (A), or 293 T cells transiently transfected with GFP or GRK5-Flag (B) were lysed, immunoprecipitated with M2 affinity gel, and analyzed by Western blotting with the indicated antibodies. (C) Endogenous DDB1 complexes were immunoprecipitated from untransfected 293 T cell lysate using the anti-DDB1 antibody and analyzed by Western blotting with GRK5 antibody. Mouse IgG was included as a control. * indicates mouse IgG.

The association of GRK5 with DDB1-CUL4 complex was confirmed by immunoprecipitation and Western analysis in MDA-MB-231 cells stably expressing GRK5-Flag and in 293 T cells transiently transfected with GRK5-Flag. DDB1, CUL4A, and CUL4B were detected in the immunocomplex of the Flag-tagged GRK5 (Fig. 2A and 2B). The RING finger protein ROC1 was also detected in GRK5 precipitates in 293 T cells (Fig. 2B) and MDA-MB-231 cells (data not shown). Damage DNA binding protein 2 (DDB2), a known binding protein of DDB1, functioning in the global genome nucleotide excision repair, was also detected in the GRK5 precipitates (Fig. 2A and Fig. 2B). Furthermore, endogenous GRK5 was detected in DDB1 immunocomplexes in 293 T cells (Fig. 2C), demonstrating association between endogenous GRK5 and endogenous DDB1 in vivo.

### DDB1 Targets GRK5 to CUL4 to Form a Complex

**Figure 3:**
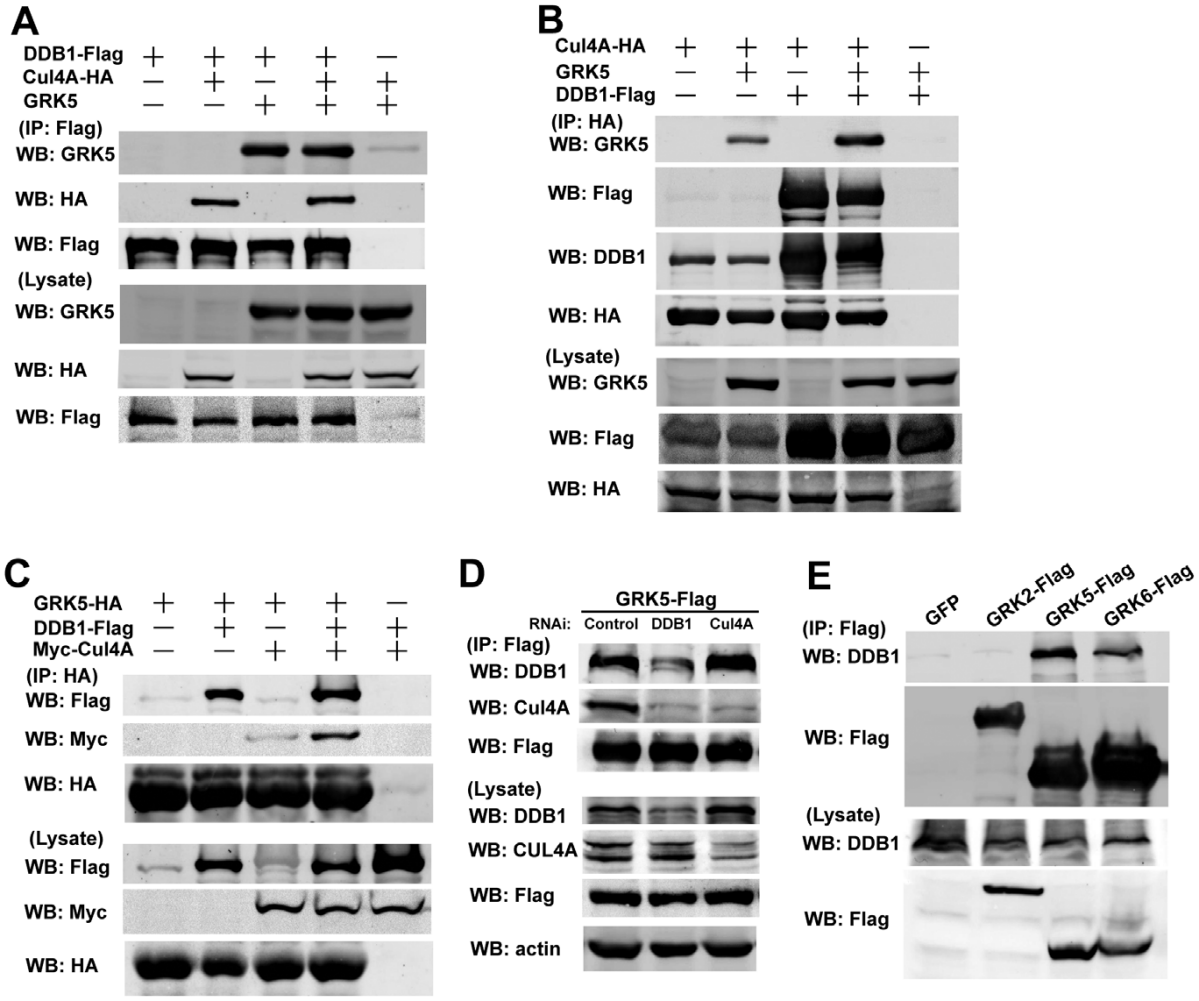
DDB1 associates with GRK5 and targets GRK5 to CUL4. (A–C) 293 T cells were transiently co-transfected with indicated plasmids alone or co-expressing both or all of them. The cell lysates were immunoprecipitated with M2 affinity gel (A) or anti-HA agarose (B and C) and then the immunoprecipitates and cell lysate input were analyzed by Western blotting with the indicated antibodies. (D) 293 T cells were co-transfected with GRK5-Flag and indicated shRNA plasmids. Cells were immunoprecipitated with M2 affinity gel. The immunoprecipitates and cell lysate input were analyzed by Western blotting with the indicated antibodies. (E) 293 T cells expressing Flag-GRK2, Flag-GRK5, Flag-GRK6, or control plasmid were lysed and immunoprecipitated with M2 affinity gel. The immunoprecipitates and the cell lysate input were analyzed by Western blotting with the indicated antibodies.

To test the idea that DDB1 acts as an adapter to recruit GRK5 to CUL4 to form a complex, epitope-tagged CUL4A, DDB1, and GRK5 were expressed in 293 T cells and protein associations were analyzed by immunoprecipitation. As shown in Fig. 3A, the association of DDB1 with GRK5 could be detected in the absence of CUL4A-HA expression, and similarly, the association of DDB1 with CUL4A-HA could occur in the absence of GRK5 expression (Fig. 3A), confirming the interaction of GRK5 and CUL4A with DDB1. When CUL4A-HA, DDB1-Flag, and GRK5 were co-expressed, all three proteins were present in a complex. However, in the absence of DDB1-Flag, the association between CUL4A-HA and GRK5 was significantly attenuated (Fig. 3B). These data suggest that DDB1 is required for the association of GRK5 with CUL4A. The basal interaction of GRK5 with CUL4A-HA in the absence of DDB1-Flag expression was likely a result from co-immunoprecipitated endogenous DDB1 by CUL4A-HA, since significant amount of endogenous DDB1 could be detected in the CUL4A immunoprecipitates (Fig. 3B, panel 3). Similar results were obtained when using GRK5-HA to immunoprecipitates DDB1 and CUL4A (Fig. 3C). Furthermore, knockdown of DDB1 significantly reduced the amount of CUL4A in GRK5 immunoprecipites. However, knockdown of CUL4A had no significant effect on the association between GRK5 and endogenous DDB1 (Fig. 3D). These results further support the notion that DDB1 acts as an adaptor to link CUL4A with GRK5.

Based on the overall structural organization and homology of GRKs, GRK family proteins can be divided into three subfamilies: GRK1 and GRK7; GRK2 and GRK3; and GRK4, GRK5, and GRK6 [13]. We examined the interaction of DDB1 with other members of GRK family proteins. As shown in Fig. 3E, DDB1 could be detected in the immunoprecipitates of GRK5-Flag as well as GRK6-Flag, but not in GRK2-Flag immunoprecipitates, suggesting the specific complex formation of DDB1 with GRK4 subfamily proteins.

### DDB1 Regulates GRK5 Ubiquitination

**Figure 4:**
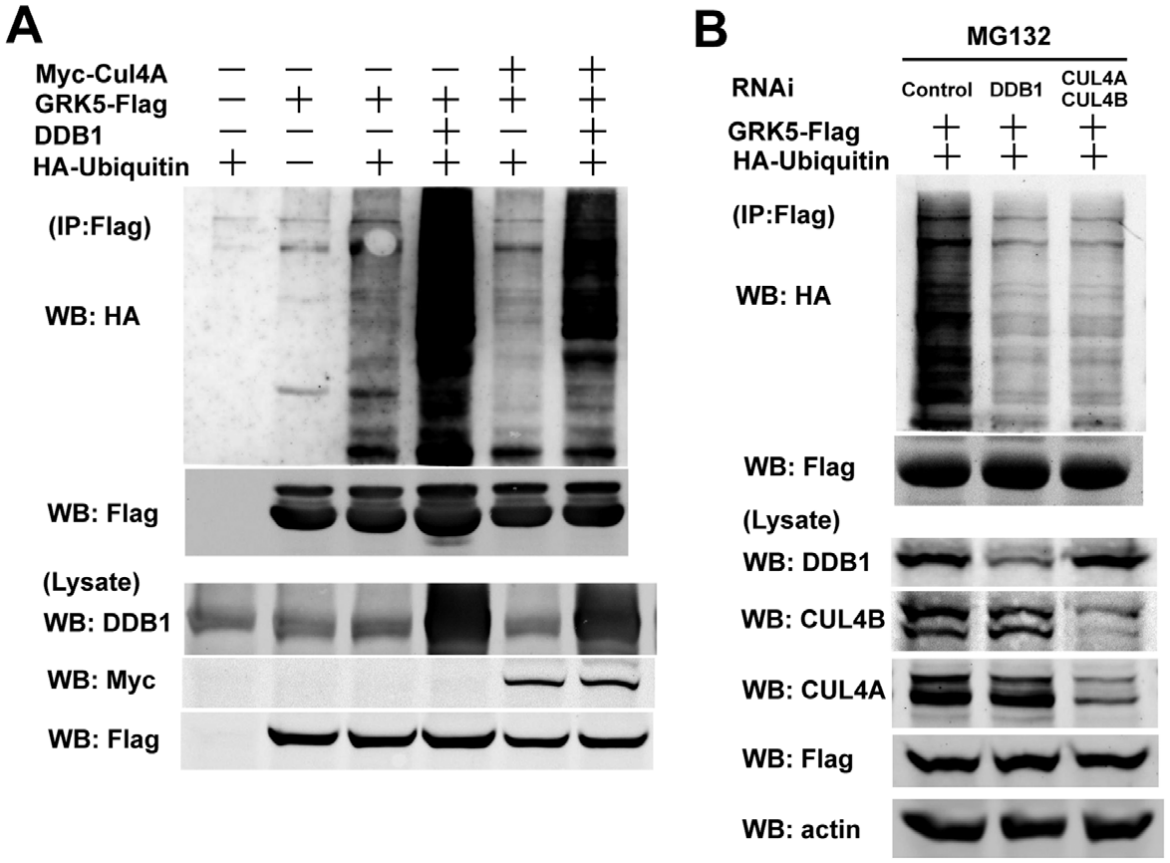
DDB1 regulates GRK5 ubiquitination. (A) 293 T cells were co-transfected with GRK5-Flag, HA-Ub, DDB1, and Myc-CUL4A in various combinations as indicated. Cell lysate was immunoprecipitated with M2 affinity gel. The GRK5-ubiquitin complex was detected with anti-HA antibody after immunoblotting. (B) 293 T cells were co-transfected with GRK5-Flag, HA-Ub, control RNAi, DDB1RNAi, CUL4A RNAi, and CUL4B RNAi in various combinations as indicated. Cells were pretreated with 10 µM MG132 for 4 h, and GRK5-ubiquitin complex was examined as in A.

Since DDB1 targets GRK5 to the CUL4 ubiquitin ligase complex, we tested whether DDB1 regulates GRK5 ubiquitination and degradation. 293 T cells were co-transfected with plasmids encoding Flag-tagged GRK5 and HA-tagged ubiquitin, in combination with control plasmid, DDB1, or myc-CUL4A. As shown in Fig. 4A, expression of DDB1 alone or co-expression of DDB1 with CUL4A significantly increased GRK5 ubiquitination, while expressing CUL4A alone did not affect the ubiquitination of GRK5. To examine the requirement of DDB1-CUL4 complex for GRK5 ubiquitination, 293 T cells were co-transfected with GRK5-Flag, HA-ubiquitin, and shRNA targeting DDB1 or CUL4. As shown in Fig. 4B, GRK5 ubiquitination was severely decreased by the depletion of either DDB1 or CUL4A/B.

### DDB1 Regulates GRK5 Degradation

**Figure 5:**
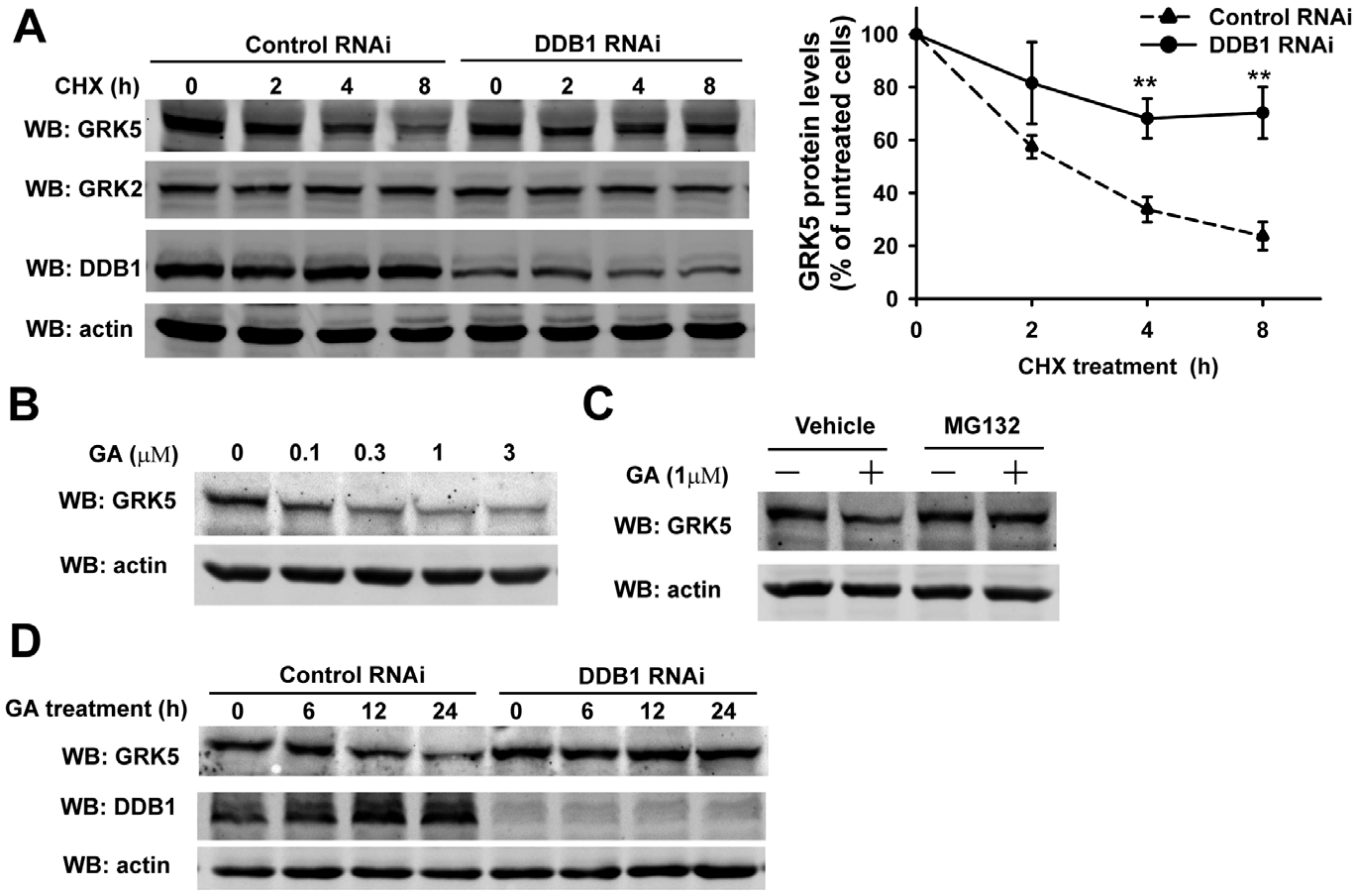
DDB1 regulates GRK5 degradation. (A) 293 T cells were infected with lentivirus expressing control RNAi or DDB1 RNAi. Cells were treated with 100 µg/ml CHX for the indicated time. Cell lysates were analyzed by Western blotting with antibodies specific for the indicated proteins. Quantitative graphs are shown. The amount of GRK5 at 0 h was defined as 100%. Data are the mean±SE from 4 independent experiments. *p<0.01. (two-way ANOVA) compared with control RNAi. (B) 293 T cells were treated with geldanamycin at indicated concentration for 24 h at 37°C. Cell lysates were analyzed by Western blotting with antibodies specific for the indicated proteins. (C) 293 T cells were pretreated with or without 10 µM MG132 for 1 h followed by incubation with geldanamycin for 12 h. Cell lysates were analyzed as in B. (D) 293 T cells were infected by lentivirus encoding control or DDB1 RNAi. Cells were treated with 1 µM geldanamycin for the indicated time. Cell lysates were analyzed as in B.

We next examined the effect of DDB1 on GRK5 degradation. 293 T cells were infected by lentivirus expressing control-shRNA or DDB1-shRNA, and the degradation of endogenous GRK5 was determined in the presence of CHX in chasing experiment. As shown in Fig. 5A, in the control cells, 50% of GRK5 was degraded in less than 3 h, whereas knockdown of DDB1 markedly attenuated the degradation of GRK5 and extended the half-life of GRK5 (∼25% reduction of GRK5 in 8 h). Hsp90 has been shown to interact with GRK2 and regulate the stability of GRK2 by preventing it from proteasome-dependent degradation [19]. A significant amount of Hsp90 peptides were also detected in the GRK5 immunoprecipitates by mass spectrometry (Fig. 1). We next examined the role of Hsp90 in regulating GRK5 stability. As shown in Fig. 5B, treatment of 293 T cells with geldanamycin, an Hsp90-specific inhibitor, significantly down-regulated the level of endogenous GRK5 in a dose-dependent manner, suggesting that Hsp90 regulates GRK5 stability. This is consistent with the previous report that treatment with geldanamycin resulted in about 80% down-regulation of GRK5 transiently expressed in COS-7 cells [19]. Furthermore, pretreatment of cells with MG132, a proteasome inhibitor, significantly inhibited geldanamycin-induced down-regulation of GRK5 (Fig. 5C), suggesting that GRK5 degradation induced by geldanamycin was predominantly through the proteasome pathway. We further examined the role of DDB1 in Hsp90 inhibition-induced degradation of GRK5. As shown in Fig. 5D, knockdown of DDB1 significantly inhibited geldanamycin-induced down-regulation of GRK5 in 293 T cells, suggesting that DDB1 mediates Hsp90 inhibition-induced proteasome-dependent degradation of GRK5.

### DDB1 Mediates UV Irradiation-induced GRK5 Degradation

**Figure 6:**
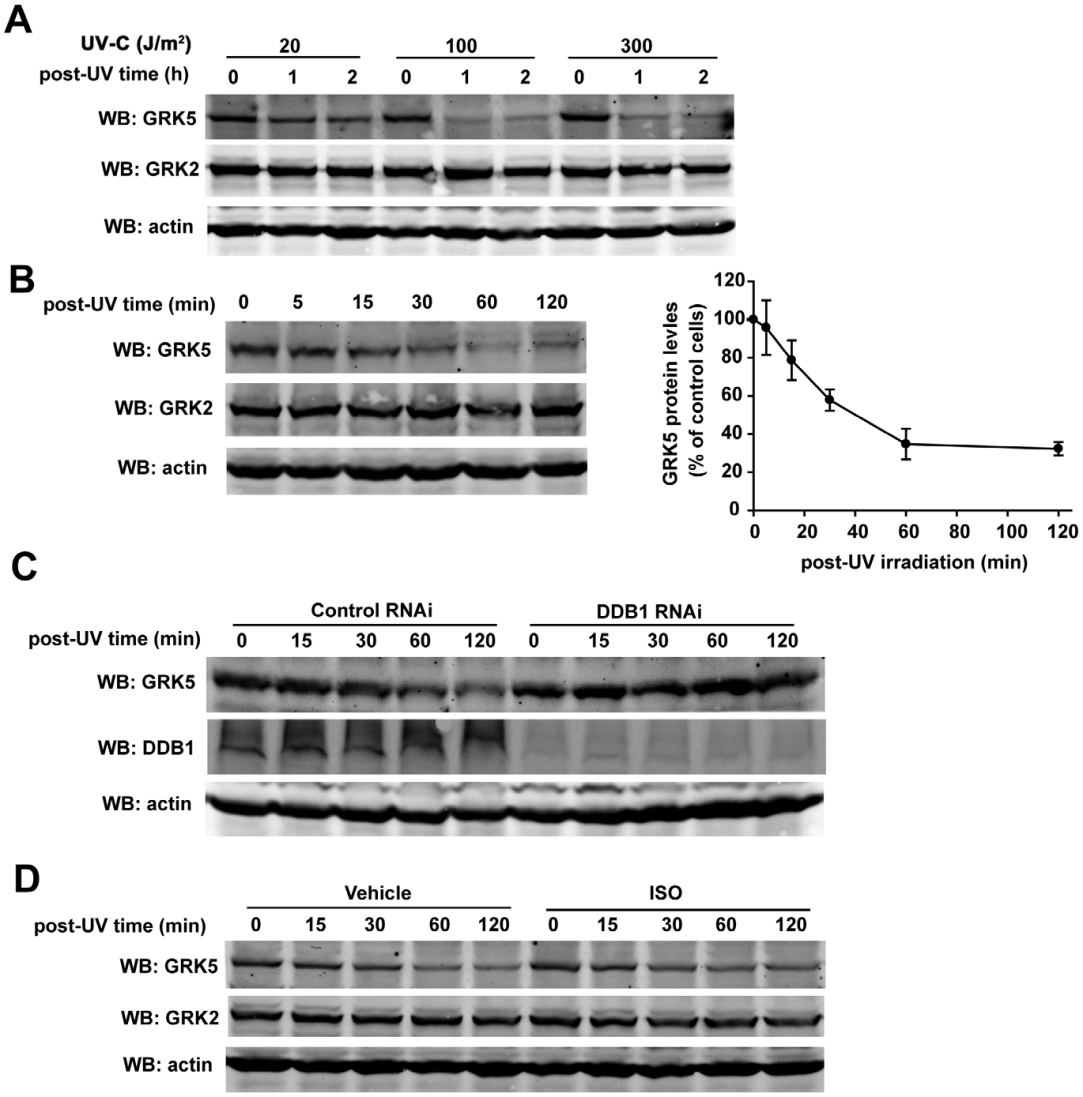
DDB1 mediates UV irradiation-induced GRK5 degradation. (A) 293 T cells were treated with 20, 100 or 300 J/m^2^ of UV-C irradiation. One or two hours after UV treatment, cells were harvested and analyzed by Western blotting with antibodies specific for the indicated proteins. (B) 293 T cells were incubated for 0, 5, 15, 30, 60, or 120 min after 20 J/m^2^ UV-C irradiation, and then cells were harvested and analyzed by Western blotting with antibodies specific for the indicated proteins. Quantitative graphs are shown. The amount of GRK5 at 0 h was defined as 100%. Data are the mean±SE from 4 independent experiments. (C) 293 T cells were infected by lentivirus encoding control or DDB1 RNAi. After 72 h, cells were treated with 20 J/m^2^ UV-C irradiation, and then incubated for the indicated time. Cells were harvested and analyzed by Western blotting. (D) 293 T cells were pretreated with or without isoproterenol (1 µM) for 20 min before 20 J/m^2^ UV-C irradiation, and then incubated in the presence or absence of isoproterenol (1 µM) for the indicated time. Cells were harvested and analyzed by Western blotting.

We further explored the potential role of the DDB1-CUL4 ubiquitin ligase in GRK5 degradation in 293 T cells. It has been shown that DDB1-CUL4 ubiquitin ligase promotes degradation of many proteins including p21 [31], [32], CDT1 [26], [33], [34] and DDB2 [24] following ultraviolet light (UV) irradiation. The response of GRK5 following DNA damage with UV irradiation was examined. The cellular GRK5 level was significantly down-regulated following UV treatment with as little as 20 J/m^2^ UV, whereas GRK2 levels remained largely unchanged under the same condition (Fig. 6A and 6B). Remarkably, knock down of DDB1 effectively prevented UV-induced GRK5 degradation, suggesting that UV irradiation-induced degradation of GRK5 is mediated by DDB1 (Fig. 6C). The effect of GPCR activation on the stability of GRK5 was also examined. As shown in Fig. 6D, treatment of cells with isoproterenol to activate endogenous β2-adrenergic receptors in 293 T cells [35], [36] had no significant effect on GRK5 protein level (data not shown) or UV irradiation-induced GRK5 degradation (Fig. 6D).

## Discussion

In the current study, a proteomic approach was used to screen GRK5 interacting proteins in MDA-MB-231 cells and HUVEC cells. Several proteins were detected in the GRK5 immunocomplex including SRRM2, MYH9, AP3D1, DDB1, Hsp90, UBTF4, NCL and STK38. Interestingly, other components of DDB1-CUL4 ubiquitin ligase complex including CUL4B, WDR22, GRWD1 and COPS7A, were also detected in the GRK5 immunocomplex in both MDA-MB-231 cells and HUVEC cells. We further provided evidence that GRK5 forms a complex with DDB1-CUL4-ROC1 E3 ubiquitin ligase and DDB1 acts as an adaptor to link GRK5 to CUL4 to form the complex. DDB1 regulates the ubiquitination and degradation of GRK5. Furthermore, depletion of DDB1 inhibited Hsp90 inhibitor-induced GRK5 destabilization and UV irradiation-induced GRK5 degradation. Thus, our immunoprecipitation-mass spectrometry data provide useful information about GRK5 interacting proteins in cells, and our results reveal DDB1 as a key regulator of GRK5 stability.

Altered GRK protein expression and/or activity have profound effects on cell signaling and physiological functions, and changed GRK expression has been observed in a variety of human disorders [13]. The mechanisms that govern GRK2 cellular levels have recently been addressed. GRK2 is rapidly degraded via the proteasome pathway. The ubiquitination and turnover of GRK2 are stimulated by β2-adrenergic receptor activation [14], through a mechanism involving GRK2 phosphorylation by c-Src or MAPK in a beta-arrestin-dependent manner [15], [16]. Mdm2 is subsequently identified as the key E3 ubiquitin ligase involved in GRK2 ubiquitination and degradation [18]. However, how the stability of other GRK subtypes is controlled remains largely unknown. In the current study, we identified DDB1-CUL4 complex as the key ubiquitin ligase responsible for GRK5 ubiquitination and degradation. Several lines of evidence support the notion that DDB1 serves as a linker to target GRK5 to DDB1-CUL4 E3 ligase for GRK5 ubiquitination and degradation. First, DDB1 was detected in GRK5 immunoprecipitates of lysates from different cell lines. Second, a pool of endogenous GRK5 and DDB1 can be found in the same molecular complex, as indicated by co-immunoprecipitation. Moreover, overexpression or knockdown of the protein reveals DDB1 is an adapter linking GRK5 to DDB1-CUL4 complex. Third, GRK5 ubiquitination and degradation is significantly impaired in DDB1/CUL4-knockdown cells. Finally, we found that degradation of GRK5 induced by both Hsp90 inhibitor and UV-irradiation could be both inhibited in DDB1 deficient cells. Thus this may serve as a new regulation mechanism for GRK5 stability. It will be interesting to further explore if the DDB1-mediated GRK5 degradation is DDB1/GRK5 interaction-dependent, or if there is a direct interaction between GRK5 and DDB1.

We also show that DDB1 preferentially associates with GRK4 family proteins. DDB1 could be observed in both GRK5 and GRK6, but not GRK2 immunoprecipitates. GRK2 has been shown to interact with Mdm2 [18]. These results suggest that different GRK subtypes may form complexes with diverse E3 ubiquitin ligases. In addition, Penela et al. reported that GRK2 has a half-life of about 1 h in C6 glioma and Jurkat cells [14], [15]. In this study, we observed that in 293 T cells, endogenous GRK5, but not GRK2, was rapidly degraded after CHX treatment. Our results are consistent with previous reports showing that the half-life of GRK2 in HL60 cells is 20–24 h [19], [37]. This may reflect difference in degradation mechanisms of GRK2 among these cell types.

Hsp90 has been shown to interact with GRK2 and GRK3, and regulates the stability of GRK2 and GRK3 [19], [38]. Inhibition of Hsp90 results in rapid, proteasome-dependent, down-regulation of GRK2 and GRK3 [19], [38]. In this study, we found that inhibition of Hsp90 resulted in proteasome-dependent degradation of GRK5. These studies and our data implicate that Hsp90-mediated stabilization and proteasome-dependent degradation may play a general role in regulation of the stability of GRK family proteins. Hsp90 has also been reported to interact with and participate in the regulation of other kinases, such as Erb2, Akt/PKB, and Raf-1 [39]. Inhibition of Hsp90 by geldanamycin results in enhanced degradation of these proteins [39]. Therefore, stabilization of client protein seems to be a general function of Hsp90. We further provide the evidence that the degradation of endogenous GRK5 induced by Hsp90 inhibition was mediated by DDB1. Depletion of DDB1 inhibited geldanamycin-induced degradation of GRK5. These results suggest that disrupting the interaction between GRK5 and Hsp90 induces DDB1-CUL4 complex-mediated ubiquitination-proteasome dependent degradation of GRK5, and put forward the notion that the stability of GRK5 was counter-regulated through interaction with DDB1-CUL4 complex and Hsp90.

DDB1-CUL4 complex controls ubiquitination and stability of many cellular substrates including p21 [31], [32], DDB2 [24], Cdt1 [26], [33], [34], p27 [40], TSC2 [41], Merlin [42], Chk1 [43], c-Jun [28], and histones [21], [44], through DDB1 bridged interaction of substrates with CUL4 E3 ubiquitin ligase. p21 and Cdt1 are the most established substrates of DDB1-CUL4 ubiquitin ligase complex after UV irradiation. The degradation of p21 and Cdt1 following UV irradiation, which promotes DNA repair and suppresses replication licensing, is critical for the cell to respond to DNA damage. Interestingly, in this study, we found that GRK5, but not GRK2, is degraded after UV irradiation, and DDB1 mediates UV irradiation-induced GRK5 degradation. The identification of GRK5 as a DDB1-CUL4 complex target following UV irradiation adds it to the growing list of cellular substrates targeted to CUL4 by DDB1. The physiological consequence of UV irradiation-induced GRK5 degradation remains to be explored.

The current study is aimed to explore the possible regulation of GRK5 by the DDB1-CUL4 ubiquitin ligase complex. However, the association of GRK5 with DDB1-CUL4 complex may also result in the phosphorylation of DDB1 and/or Cul4 by GRK5, and thus regulates the function of DDB1-CUL4 ubiquitin ligase complex itself. In fact, cells deficient of GRK5 or DDB1 exhibit similar phenotypes, such as apoptosis and cell growth retardation [8], [45], [46]. It would be interesting to determine whether GRK5 regulates the function of DDB1-CUL4 ubiquitin ligase complex and the ubiquitination of other substrates of DDB1-CUL4 complex, and investigate whether DDB1-CUL4 complex is involved in GRK5 deficiency-induced cell growth retardation in future studies.

We also identified other new interacting proteins of GRK5, including SRRM2 and STK38, by immunoprecipitation-mass spectrometry in MDA-MB-231 cells. SRRM2, which has been shown to be involved in pre-mRNA splicing [47], [48], is the most abundant among proteins identified by mass spectrometry in GRK5-immunocomplex in both MDA-MB-231 cells and HUVEC cells (supplemental Table S1 and S2). These results implicate possible function of GRK5 in mRNA splicing. Another interesting interacting protein of GRK5 is STK38 which is identified in GRK5 immunocomplex in cancer cell lines (MDA-MB-231 cells), but not in normal cells (HUVEC). STK38, also known as NDR1, is a serine-threonine protein kinase of AGC kinase subfamily, it has been shown to regulate many essential processes including cell division [49], proliferation [49], centrosome duplication [50] and apoptosis [51]. Interestingly, GRK5 has also been shown to be involved in proliferation and apoptosis [8], [46], [52]. It will be of interest to examine the possible role of interaction between GRK5 and STK38 in regulating these processes.

## Materials and Methods

### Reagents and Antibodies

Dulbecco’s modified Eagle’s and Leibovitz’s L-15 media were purchased from Invitrogen. Fetal bovine serum (FBS) was obtained from Hyclone. Mouse anti-DDB1 antibody, rabbit anti-ROC1 antibody, and mouse antibody against Myc epitope were from Invitrogen. Rabbit anti-DDB2 antibody, rabbit anti-GRK2 antibody, and protein A/G agarose were from Santa Cruz Biotechnology. Rabbit anti-CUL4B antibody, rabbit anti-β-actin antibody, mouse antibody against FLAG epitope, rabbit antibody against HA epitope, mouse IgG, anti-Flag M2 affinity gel, and anti-HA agarose were from Sigma. Goat anti-GRK5 antibody was from R&D. Geldanamycin and cycloheximide (CHX) were from Sigma.

### Plasmid Construction

Plasmids encoding tagged bovine GRK2 and GRK5, and human GRK6 were constructed by PCR mutagenesis. Construction of shRNA plasmids for human DDB1, human CUL4A, and human CUL4B was performed as described [53]. LacZ shRNA was used as a control. The shRNA lentivirus system, which uses the FG12 and package vector to enable simultaneous expression of the GFP protein and shRNA were obtained from Dr. Gang Pei (Chinese Academy of Sciences, Shanghai, China). Sequences for DDB1 RNAi are: GCG AGA GCA TTG ACA TCA TTA (sense) and TAA TGA TGT CAA TGC TCT CGC (antisense). CUL4A: GCA GGA CCA CTG CAG ACA AAT (sense) and ATT TGT CTG CAG TGG TCC TGC (antisense). CUL4B: GCC ACG TAC CGA TAC AGA AGA (sense) and TCT TCT GTA TCG GTA CGT GGC (antisense). The plasmids DDB1-Flag (Addgene plasmid 19918), CUL4A-HA (Addgene plasmid 19907), and Myc-CUL4A (Addgene plasmid 19951) were gifts of Dr. Yue Xiong. The plasmid DDB1 was constructed into pcDNA3.0.

### Cell Culture and Plasmid Transfection

293 T cells were cultured in Dulbecco’s modified Eagle’s medium containing 10% FBS. Cells were seeded in 60 or 100 mm tissue culture dishes at 0.6–2×10^6^/dish 20 h before transfection and transfected plasmid using calcium phosphate/DNA co-precipitation method or infected by lentivirus. Assays were performed 48 h (for expressing experiments) or 72 h (for RNAi experiments) after transfection. MDA-MB-231 cells stably expressing GFP or GRK5-Flag were cultured in Leibovitz’s L-15 plus 10% FBS. For protein decay analysis, 293 T cells were treated with CHX at 100 µg/ml.

### Generation and Titer of Lentivirus

The sequences predicted to target the human DDB1 or CUL4A were used for the construction of shRNA lentivirus. FG12-hU6-shRNA and packaging vectors were co-transfected into 293 T cells and the resulting supernatant were collected after 48 h. Virus was recovered after ultracentrifugation resuspension in phosphate-buffered saline. Titers were determined by infecting 293 T cells with serial dilutions of concentrated lentivirus. GFP expression in infected cells was determined 48 h after infection, and for a typical preparation, the titer of lentivirus was approximately 10^8^ infectious units (IFU)/ml.

### Isolation and Identification of GRK5-associated Proteins

MDA-MB-231 cells were stably transfected with GRK5-Flag or GFP lentivirus. HUVEC cells were transiently transfected with GRK5-Flag or GFP by lentivirus. About 10^8^ cells were harvested and lysed in IP buffer (50 mM Tris.HCl, pH 7.5, 150 mM NaCl, 0.5% NP-40, 10% glycerol, 1 mM EDTA, 10 mM NaF, plus 10 µg/ml aprotinin, 10 µg/ml benzamidine, and 0.2 mM PMSF) for 2 h. The lysate was centrifuged and the supernatant was incubated with anti-FLAG M2 affinity gel at 4°C for 8 h. The beads were subsequently washed. Bound proteins were eluted by addition of SDS sample buffer. The resulting protein complexes were electrophoresed on a 4–20% gradient polyacrylamide gel and revealed by staining with Coomassie Blue. Six selected bands were excised from the gel and analyzed by mass spectrometry. The corresponding GFP control lane were also excised and analyzed by mass spectrometry. The mass spectrometry analysis was performed at the Shanghai Applied Protein Technology Co.Ltd.

### LC-MS/MS

Gel pieces were destained with 30% ACN/100 mM NH_4_HCO_3_ and dried in a vacuum centrifuge. The in-gel proteins were reduced with dithiothreitol (10 mM DTT/100 mM NH_4_HCO_3_ ) for 30 minutes at 56°C, then alkylated with iodoacetamide (50 mM IAA/100 mM NH_4_HCO_3_) in the dark at room temperature for 30 minutes. Gel pieces were briefly rinsed with 100 mM NH_4_HCO_3_ and ACN, respectively. Gel pieces were digested overnight in 12.5 ng/mL trypsin in 25 mM NH_4_HCO_3_. The peptides were extracted three times with 60% ACN/0.1% TFA. The extracts were pooled and dried. EttanTM MDLC system (GE Healthcare) was applied for desalting and separation of tryptic peptides mixtures. In this system, samples were desalted on RP trap columns (Agilent Technologies), and then separated on a RP column (Column technology Inc.). Mobile phase A (0.1% formic acid in HPLC-grade water) and the mobile phase B (0.1% formic acid in acetonitrile) were selected. 20 µg of tryptic peptide mixtures was loaded onto the columns, and separation was done at a flow rate of 2 µL/min by using a linear gradient of 4–50% B for 30 min. A LTQ VELOS (Thermo Electron) equipped with an electrospray interface was connected to the LC setup for eluted peptides detection. Data-dependent MS/MS spectra were obtained simultaneously. Each scan cycle consisted of one full MS scan in profile mode followed by five MS/MS scans in centroid mode with the following Dynamic ExclusionTM settings: repeat count 2, repeat duration 30 s, exclusion duration 90 s. Each sample was analyzed in triplicate. MS/MS spectra were automatically searched against the non-redundant International Protein Index (IPI) human protein database (version 3.53) using the BioworksBrowser rev. 3.1(Thermo Electron).

### Immunoprecipitation and Western Blotting

Cells were washed with ice-cold PBS and lysed in IP buffer for 1.5 h as described [54]. The lysate was centrifuged and the supernatant was incubated with anti-FLAG M2 affinity gel or anti-HA agarose at 4°C for 8 h. The beads were subsequently washed, and the proteins bound to the beads were eluted. The samples were detected in the subsequent Western procedures with the corresponding antibody. Blots were incubated with IRDye 800CW-conjugated or 700CW-conjugated antibody (Rockland Biosciences) and infrared fluorescence images were obtained with the Odyssey infrared imaging system (Li-Cor Bioscience).

### Ubiquitination Assay

293 T cells seeded in 60 mm dishes were transfected with different combinations of expression vectors for HA-ubiquitin, GRK5-Flag, and other indicated plasmids. Cells were lysed in ubiqutination buffer (50 mM HEPES, pH 8.0, 250 mM NaCl, 0.5% NP-40, 10% glycerol, 2 mM EDTA, 20 mM NaF, plus 10 µg/ml aprotinin, 10 µg/ml benzamidine, and 0.2 mM PMSF). Cell lysate was immunoprecipitated with anti-Flag M2 affinity gel and analyzed by immunobloting with anti-HA antibody.

### CHX Treatment

For protein decay analysis, 293 T cells were infected by LacZ RNAi or DDB1 RNAi lentivirus for 48 h and then reseeded onto 60 mm culture dish at 7×10^5^ cells per dish. After 40 h, cells were exchanged with fresh culture medium and CHX (100 µg/ml) was added at the proper time point for CHX treatment for 0 h, 2 h, 4 h, or 8 h. After CHX treatment, cells were washed with PBS, and total cell lysates were prepared for Western blotting.

### UV-C Irradiation

UV-C (254 nm) irradiation was performed with a UV-C transilluminator (Hoefer UVC 500). The culture medium was replaced with complete medium two hours before the UV-C treatment. Cells were irradiated at 80% confluency with a 20 J/m^2^ dose or indicated dose after removal of the medium in the 60 mm petri dish without the lid. After irradiation, cells were post-incubated in their culture medium for the indicated time and were lysed directly in RIPA^+^ buffer (50 mM Tris.HCl, pH 8.0, 150 mM NaCl, 1% NP-40, 0.5% Deoxycholic Acid, 0.1% SDS, 5 mM EDTA, 10 mM NaF, 10 mM Disodium pyrophosphate, plus 10 µg/ml aprotinin, 10 µg/ml benzamidine, and 0.2 mM PMSF), and equal amount of protein was loaded on gels.

### Statistical Analysis

Data were analyzed using two-way ANOVA for comparison of independent means with pooled estimates of common variances.

## Supporting Information

Table S1 **List of proteins associated with GRK5 identified by mass spectrometry in MDA-MB-231 cells.** The GRK5 immunocomplex isolated from MDA-MB-231 cells stably expressing GFP or GRK5-Flag were separated on SDS-PAGE and stained with Coomassie Blue. Six pieces of gels (250∼400 kDa, 200∼250 kDa, 75∼200 kDa, 45∼60 kDa, 25∼30 kDa, 15∼20 kDa) were excised and labled as line 1-line 6 as indicate in Fig. 1A. The corresponding control lane was also excised. These gels were analyzed by mass spectrometry. The peptide counts of proteins obtained in GRK5 immunocomplex in MDA-MB-231 cells by mass spectrometry were recorded. Proteins detected in both the control lane and GRK5-immuocomplex lane were excluded from the table. We also provide the peptide counts of proteins identified in HUVEC cells for comparison. The proteins obtained in HUVEC cells but not in MDA-MB-231 cells by mass spectrometry were not listed in the Table S1.
(XLSX)

Table S2 **List of proteins associated with GRK5 identified by mass spectrometry in HUVEC cells.** The GRK5 immunocomplex isolated from HUVEC cells transiently transfected with GFP or GRK5-Flag were treated as above. The peptide counts of proteins obtained in GRK5 immunocomplex in HUVEC cells by mass spectrometry were recorded. Proteins detected in both the control lane and GRK5-immuocomplex lane were excluded from the table.
(XLSX)

Table S3 **List of protein peptides in GRK5 immunocomplex identified by mass spectrometry in MDA-MB-231 cells.** The peptides of proteins obtained in GRK5 immunocomplex in MDA-MB-231 cells by mass spectrometry were recorded.
(XLSX)

Table S4 **List of protein peptides in GRK5 immunocomplex identified by mass spectrometry in HUVEC cells.** The peptides of proteins obtained in GRK5 immunocomplex in HUVEC cells by mass spectrometry were recorded.
(XLSX)
